# Evaluation for inherited and acquired prothrombotic defects predisposing to symptomatic thromboembolism in children with acute lymphoblastic leukemia: a protocol for a prospective, observational, cohort study

**DOI:** 10.1186/s12885-017-3306-5

**Published:** 2017-05-04

**Authors:** Uma H. Athale, Caroline Laverdiere, Trishana Nayiager, Yves-Line Delva, Gary Foster, Lehana Thabane, Anthony KC Chan

**Affiliations:** 1 Division of Hematology/ Oncology, McMaster Children’s Hospital, Hamilton Health Sciences, 1280 Main Street West, Room HSC 3N27, Hamilton, ON L8S 4K1 Canada; 2 Department of Pediatrics, McMaster University, 1280 Main Street West, Hamilton, ON L8S 4K1 Canada; 3 Department of Pediatrics, Hematology Oncology Service, CHU Ste-Justine, University of Montréal, 3175, Côtes-Sainte-Catherine, Montréal, QC H3T 1C5 Canada; 4 Department of Clinical Epidemiology and Biostatistics, McMaster University, 50 Charlton Ave. E, Hamilton, Canada

**Keywords:** Leukemia, Children, Thromboembolism, Chemotherapy, Prothrombotic defects

## Abstract

**Background:**

Thromboembolism (TE) is a serious complication in children with acute lymphoblastic leukemia (ALL). The incidence of symptomatic thromboembolism is as high as 14% and case fatality rate of ~15%. Further, development of thromboembolism interferes with the scheduled chemotherapy with potential impact on cure rates. The exact pathogenesis of ALL-associated thromboembolism is unknown. Concomitant administration of asparaginase and steroids, two important anti-leukemic agents, is shown to increase the risk of ALL-associated TE. Dana-Farber Cancer Institute (DFCI) ALL studies reported ~10% incidence of thrombosis with significantly increased risk in older children (≥10 yrs.) and those with high-risk ALL. The majority (90%) of thromboembolic events occurred in the Consolidation phase of therapy with concomitant asparaginase and steroids when high-risk patients (including all older patients) receive higher dose steroids. Certain inherited and acquired prothrombotic defects are known to contribute to the development of TE. German investigators documented ~50% incidence of TE during therapy with concomitant asparaginase and steroids, in children with at least one prothrombotic defect. However, current evidence regarding the role of prothrombotic defects in the development of ALL-associated TE is contradictory. Although thromboprophylaxis can prevent thromboembolism, ALL and it’s therapy can increase the risk of bleeding. For judicious use of thromboprophylaxis, identifying a population at high risk for TE is important. The risk factors, including prothrombotic defects, predisposing to thrombosis in children with ALL have not been defined.

**Methods:**

This prospective, observational cohort study aims to evaluate the prevalence of inherited prothrombotic defects in children with ALL treated on DFCI 05–01 protocol and the causal relationship of prothrombotic defects in combination with patient and disease-related factors to the development of TE. We hypothesize that the combination of prothrombotic defects and the intensive therapy with concomitant high dose steroids and asparaginase increases the risk of TE in older patients and patients with high-risk ALL.

**Discussion:**

The results of the proposed study will help design studies of prophylactic anticoagulant therapy. Thromboprophylaxis given to a targeted population will likely reduce the incidence of TE in children with ALL and ultimately improve their quality of life and prospects for cure.

## Background

Acute lymphoblastic leukemia (ALL), the most common cancer in children, is now curable in over 80% of the children with current aggressive therapy [1, 2]. However, such therapy is associated with significant, sometimes fatal, complications. These therapy-related morbidity and mortality can limit the dose intensification of antileukemic agents and compromise the prospects of cure [3]. Thus, to improve the cure-rates and quality of life of children with ALL, it is important to reduce specific, avoidable therapy-related complications.

Thromboembolism (TE) is one such serious complication in association with ALL therapy in children [4, 5]. Overall TE is rare in general pediatric population with ~0.19 events per 10,000 children [6–8]. In contrast, children with ALL are at much higher risk for TE; reported incidence of symptomatic TE varies from 1% to 14% and that for asymptomatic TE is up to 37% [4, 5, 9]. The majority of the symptomatic TE occur in potentially fatal sites, ***~***50% in the central nervous system (CNS), 2% pulmonary embolism (PE) and 2% in the right atrium [4, 5]. TE including CNS-TE is associated with significant morbidity. In addition, development of TE interferes with the scheduled ALL-therapy; such interruptions are known to compromise cure rates [5, 10]. The average case fatality ratio from TE in children with ALL is 15% [5]. With ~15–20% all-cause mortality in children with ALL; TE may be an important cause of death during ALL-therapy [5, 11].

### Pathogenesis of thromboembolism in children with acute lymphoblastic leukemia

**Fig. 1:**
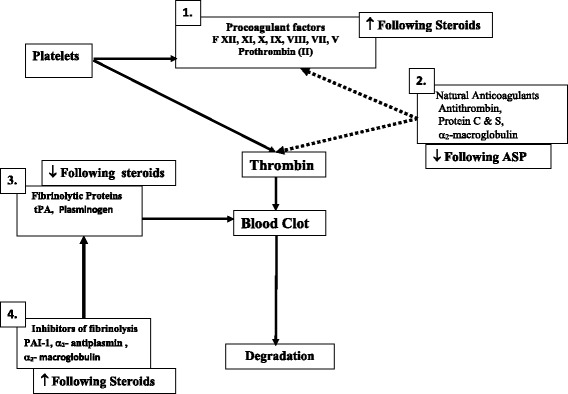
Abbreviations: ASP. Asparaginase; tPA, tissue plasminogen activator; PAI1, plasminogen activator inhibitor 1; Thrombin activation is the central mechanism of hemostasis. Under physiological conditions, blood is maintained in the fluid state by a delicate balance between the pro-coagulant factors [1], natural anti-coagulants [2], and fibrinolytic system which consists of fibrinolytic proteins [3] and inhibitors of fibrinolysis [4]. Thus, an increase in the levels of procoagulant factors combined with reduction in natural anticoagulants or fibrinolytic potential may result in predisposition for thrombosis. ASP and steroids act on different hemostasis pathways as shown above

ALL-associated TE is a multifactorial entity [5, 12, 13]***.*** Leukemia, its therapy, and factors inherent to the host seem to collectively contribute to the risk of thrombosis in children with ALL. Central venous line (CVL), a well-known risk factor for TE, is commonly used in children with ALL [4, 5, 9].Children with ALL have evidence of thrombin activation at diagnosis as well as during first several months of therapy [4, 5, 12, 14–20]. Thrombin generation is the central event in the blood clot formation. Figure 1 depicts the role of thrombin in clot formation and possible factors affecting the thrombin generation in association with ALL. Asparaginase (ASP) and steroids form the backbone of most frontline ALL therapy protocols***.*** Available evidence indicate that ASP and steroids induce an acquired prothrombotic state by affecting different hemostatic pathways (outlined in Fig. 1) [12, 21]. ASP, a bacterially derived enzyme, leads to rapid depletion of extracellular pools of asparagine in the body; the resultant inhibition of protein synthesis is responsible for major toxicities of ASP therapy including haemostatic abnormalities [22]. ASP-therapy is shown to causes suppression of natural anticoagulants [antithrombin (AT), protein C (PC) and protein S (PS)] and this reduction, especially AT, is mainly responsible for the ASP-associated prothrombotic state [14, 21].

Steroids are shown to increase coagulation factors II and VIII, and to induce a hypofibrinolytic state with elevation of plasminogen activator inhibitor 1 (PAI1) levels and reduction of α_2_-macroglobulin and tissue plasminogen activator [14, 23–35]. Animal studies have shown a dose dependent effect of steroids on fibrinolytic system [36, 37]. Recent studies showed that concomitant administration of ASP with steroids increases the risk of TE in patients with ALL compared to temporally separate use of ASP and steroids [38–40].

### Effect of congenital or acquired prothrombotic defects in the development of ALL-associated TE

Certain prothrombotic defects increase the risk of TE in adults and children. These include Factor V Leiden (FVL), deficiency of natural anticoagulants (PC, PS and AT), and elevated levels of coagulation factors VIII, IX and XI, [13, 41–43]. In addition, mutations of prothrombin (PT) gene G20210A and methylene tetrahydrofolate reductase (MTHFR) C677T are common and mild risk factors for venous TE in general population [44, 45]. Elevated levels of homocystein (Hcy) and lipoprotein (a) [Lp (a)] are recently identified risk factors for TE [13, 46, 47]. These inherent host factors in the presence of other associated risk factors can increase the risk of TE especially in children with ALL (Table 1) [43]. A multi-center, prospective study of German children receiving therapy on Berlin-Frankfurt-Münster (BFM) ALL protocol 90/95 showed that 48.5% (27/58) children with at least one prothrombotic defect developed venous TE compared to 2.2% (5/231) children without any identified prothrombotic defect (*p* < 0.001) [38].

**Table 1:** Potential interactions of thrombophilia and antileukemic agents

Thrombophilia	ALL or Chemotherapeutic agent	Possible interaction
PT gene polymorphism 20210A	ALL	PT mutation may exaggerate ALL-induced thrombin generation
	Corticosteroid	May induce higher levels of PT
MTHFR C677T	Methotrexate	By inhibiting folate pathway induces functional MTHFR deficiency even in heterozygous patients with resultant high Hcy levels
FVL	Asparaginase	By reducing protein C levels may exaggerate the effects of FVL even in heterozygous subjects
Protein C, S and AT deficiency	Asparaginase	By inhibiting protein synthesis results in reduction in Proteins C, S and AT
Elevated pro-coagulant factors VIII:C, IX and XI	Corticosteroid	May induce higher levels of factors VIII:C, IX and XI
Elevated Lp(a) levels	Asparaginase	Lead to mark elevation in Lp(a)

*Abbreviations*: *ALL* acute lymphoblastic leukemia, *PT* prothrombin, *MTHFR* methylene tetrahydrofolate reductase, *Hcy* homocysteine, *FVL* Factor V Leiden, *AT* antithrombin, *FVIII:C* Coagulation factor VIII:C, *Lp(a)* Lipoprotein a

By alterations in hemostatic proteins ALL and it’s therapy may exacerbate the deleterious effects of inherent thrombophilia, even for those factors which otherwise pose mild risk for TE in general population (namely PT and MTHFR mutations). Table 1 outlines the potential interaction of inherent thrombophilia with ALL and commonly used antileukemic agents.

Although association of antiphospoholipid antibodies (APLA) with TE is well known, it is not very well studied in children with ALL. In a multicenter, prospective Prophylactic Antithrombin Replacement in Kids with Acute Lymphoblastic Leukemia Treated with Asparaginase (PARKAA) study, 8 of 60 (13%) children with ALL had APLA; four of them developed CVL related TE [48]. Although small, this study highlights the potential importance of thrombotic risk posed by APLA in children with ALL.

### Thrombophilia as a risk factor for ALL-associate TE

Certain ethnic groups seem to have high prevalence of thrombophilia; 43% of Arab and Jewish children with ALL are reported to have inherited thrombophilia [49]. Very few studies have evaluated prevalence of thrombophilia in children with ALL [38, 39, 48–50] and only 3 studies evaluated the impact of thrombophilia on the development of TE [38, 39, 48]. These studies reported wide variability in prevalence of thrombophilia (18% to 40%) and frequency of TE in children with ALL and thrombophilia (0 to 48%); probably related to the different ethnicity of the population studied and marked variability in the extent of thrombophilia tested. Further, use of different therapy protocols and small sample size make it difficult to interpret the data.

Since the prevalence of thrombophilia varies with ethnicity and different therapy protocols are likely to have different effects on the hemostatic system, the results of the German studies cannot be generalized to North American children treated on different ALL protocols [38, 39].The prevalence of thrombophilia and its relationship to symptomatic TE in North American children with ALL is largely unknown.

### Relevance and importance of the proposed study: preliminary results of DFCI studies

TE is a significant problem in children receiving therapy on DFCI ALL protocols. Pilot data from Canadian institutions showed 11% prevalence of symptomatic TE in children receiving therapy on DFCI ALL protocols [51]. Older age (≥ 10-years) and high-risk (HR) disease are important risk factors for development of TE; older patients (≥ 10-years) compared to younger patients (44% vs. 4%, *p* < 0.0001) and patients with HR ALL compared to standard-risk (SR) ALL (26% vs. 2%) had higher prevalence of TE (*n* = 91) [51].

The effect of older age and HR ALL on the risk of symptomatic TE was confirmed in Consortium-wide review of earlier protocols; overall incidence of TE in children ≥10 years was 12% compared to 2% in children <10 years of age (*p* < 0.0001) [DFCI ALL studies 91–01 and 95–01 (*n* = 906)] and in children with HR ALL was 17% compared to 1.5% in children with SR ALL (*p* = 0.005) [DFCI 20–01(*n* = 118)]. Of note, data on TE was not consistently collected on earlier DFCI protocols.

The etiology of the increased susceptibility of older and HR ALL patients to TE is unknown, it is likely related to the therapy they receive. Majority of the episodes of TE in patients treated on DFCI ALL therapy protocols occurred during Consolidation phase where ASP and steroids are given concomitantly. Moreover during this phase, HR patients (including all patients ≥10 years) receive thrice as much steroids as SR patients [52]. Compared to the contemporary therapy protocols, DFCI ALL protocols use higher cumulative doses of steroids and ASP.

In contrast to BFM studies, our data showed that the dose, but not the type, of steroid used with ASP significantly altered the risk of TE on DFCI ALL-protocols. Patients receiving high-dose steroids were at significantly higher risk of TE (18.2% Vs 2.7% in patients with lower dose steroids; *p* = 0.004) [53, 54].

Our preliminary studies indicate that children who develop TE are more likely to have adverse outcome from ALL compared to those who do not develop TE. Silverman et al., on DFCI 91–01 study showed that early (< 25 weeks) discontinuation of ASP-therapy adversely affected 5-year event free survival (EFS) (73% vs. 90%; *p* < 0.01); CNS event or non-CNS-TE was responsible for early discontinuation of ASP in 20% of patients [10]. We observed 27% (3/11) mortality in children with ALL and TE compared to 6% (5/84) in children with ALL without TE (*p* = 0.048; 95% CI -5.5, 48.1).

There is no information about thrombophilia in patients treated on DFCI studies. At McMaster University, 10 of 12 patients with ALL and symptomatic TE were evaluated for prothrombotic defects; 9 had abnormal thrombophilia profile and 6 patients had >1 defect. Nine patients had increased levels of factor VIII:C (mean 3.2 U/mL; range 2.11–5.8); 3 patients had elevated fasting Lp (a) levels, one each had increased fasting Hcy level, reduced PS levels, AT deficiency and one was heterozygous for MTHFR C677T. Although the sample size is small and the prothrombotic work-up was performed after the detection of TE, this data strongly support detail evaluation of inherited and acquired prothrombotic defects as a potential risk factor for ALL-associated TE.

In summary, TE is a significant complication in children with ALL. Prothrombotic defects are shown to be prevalent in ~20% of children with ALL. Leukemia and its therapy can potentially exacerbate the deleterious effects of prothrombotic defects even in heterozygous individuals. However, the extent of the risk, if any, predisposed by prothrombotic defects in the development of TE in children with ALL (especially in relation with ALL-therapy) is unknown. Hence we propose a thrombophilia study within the context of DFCI ALL 05–01 randomized controlled trial (RCT). The proposed thrombophilia study will evaluate the role of prothrombotic defects in the development of TE as well as the interaction, if any, of these defects with patient (e.g. age), disease (e.g. risk-categorization) and therapy (e.g. the type of ASP) variables.

## Methods/trial design

### Scientific questions

The primary question is do identified congenital and acquired prothrombotic defects increase the risk of symptomatic TE in children with ALL receiving therapy on DFCI ALL 05–01 protocol? The secondary question is what other baseline and time-dependent factors increase the risk of clinically symptomatic TE in children with ALL receiving therapy according to DFCI ALL 05–01 protocol?

### Hypotheses

Primary hypothesis is that children with one or more prothrombotic defect/s are at increased risk for development of symptomatic TE during ALL-therapy on DFCI ALL 05–01 protocol compared to those without any identifiable prothrombotic defect. Secondary hypothesis is that older age of the patient (compared to younger age), HR or very high risk (VHR) ALL (compared to SR ALL), and therapy with *E. coli* ASP (compared to Pegylated (PEG) ASP), either alone or in combination with one or more prothrombotic defect, increase the risk of symptomatic TE in children on DFCI 05–01 ALL-therapy protocol.

### Overall objective

To explore the relationship of inherited and acquired prothrombotic defects with the development of symptomatic TE in children with ALL treated on DFCI ALL 05–01 protocol.

### Specific aims

#### Primary Aim

To compare the risk of development of symptomatic TE in children **with** or **without** prothrombotic defect receiving therapy on DFCI ALL 05–01 protocol.

#### Secondary Aims

1. To evaluate the effect of age of the patient (≥ 10 years versus <10 years), risk categorization of ALL (HR/VHR ALL versus SR ALL), baseline laboratory features (white blood cell counts, blast count and platelet count), type of ASP used (*E. coli* versus PEG), phase of therapy (Consolidation II versus other), either alone or in combination with any known prothrombotic defect/s, on the risk of development of symptomatic TE in children receiving therapy on DFCI ALL 05–01 protocol. (Since almost all our patients have CVL, we will not be able to evaluate the effect of CVL in relation with other factors).
2. To develop a predictive model to identify children with ALL at high risk for TE
3. To determine the prevalence of inherited and acquired prothrombotic defects in children newly diagnosed with ALL

### Research design

**Fig. 2:**
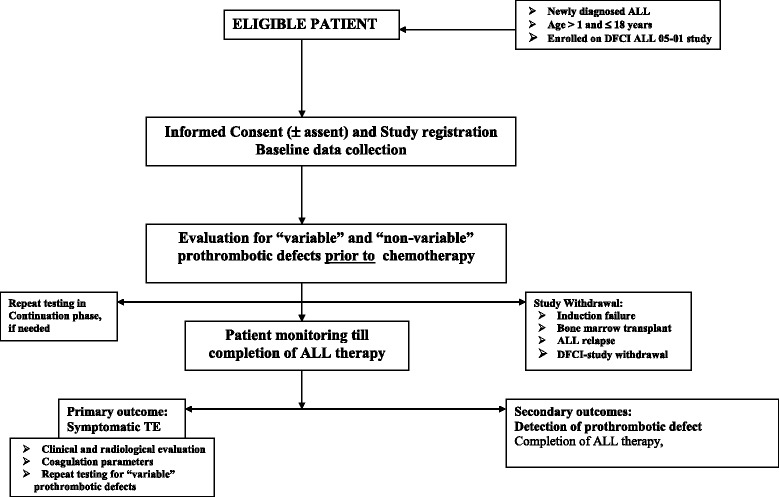
Overview of the study and patient flow

This is a prospective analytical cohort study conducted within the context of DFCI ALL randomized controlled trial 05–001. Figure 2 outlines the design of proposed thrombophilia study. DFCI ALL 05–001 study was opened for patient enrollment in three Canadian and six US institutions in June 2005. The DFCI 05-001RCT aims to compare the efficacy and toxicity of two ASP preparations namely PEG and *E. coli* ASP. The detail therapy plan and ALL risk category is described previously [55].

### Patient population

#### Study sites

This study was planned to be conducted at three tertiary care pediatric oncology centers within DFCI Consortium. However due to logistic issues the third site could not join and the study was activated at two sites: McMaster University, Hamilton and Hospital Ste. Justine, Montreal.

#### Patient eligibility

Inclusion Criteria: All children (between ages 1 to 18 years) newly diagnosed with ALL at the participating institutions and enrolled on DFCI ALL 05–01 study were eligible for the proposed study.

Children <12 months and >18 years of age and those with relapsed ALL (since they are not eligible for DFCI 05–01 study) and patients unable, or unwilling, to provide written informed consent (and/or assent) were excluded from the proposed thrombophilia study.

#### Procedure for patient identification and obtaining consent

Newly diagnosed ALL patients were identified through Pediatric Oncology service. Study staff reviewed patients’ records to determine eligibility for the proposed study. Eligible patients were approached, prior to starting ALL therapy, for informed consent. Reasons for non-participation were recorded for all screened patients.

### Observations

#### Dependent variable

Development of symptomatic TE in any location while receiving therapy on DFCI ALL 05–01 study. *Symptomatic TE* is defined as any objectively confirmed arterial or venous TE which was discovered as a result of investigations prompted by typical clinical symptoms (as outlined in Table 2). Screening for *asymptomatic* TE was not performed.

**Table 2:** Definition of symptomatic TE and preferred diagnostic evaluation

	Site	Likely clinical signs and symptoms	Diagnostic method/s
CNS	Arterial ischemic stroke +/− hemorrhage	Unexplained headaches, vomiting, visual problems, or neurological deficits, seizure, drowsiness or any change in mental status	MRI/MRA Angiogram
	Sinovenous thrombosis (SVT)		MRI /MRV CT venogram
PE	Pulmonary vasculature	Respiratory problems (shortness of breath, tachypnea, dyspnea) hypoxia, chest pain, syncope “Unexplained pneumonia”	V/Q scan Spiral CT Pulmonary angiogram
DVT	Upper venous system	Swelling, pain, tenderness, erythema, dilated vessels	Bilateral venogram is “gold standard” for diagnosis especially for subclavian/brachial vessels ^a^Doppler USG sufficient for jugular vein MRV Recommend ECHO to evaluate RA
	Lower venous system		^a^Doppler USG to evaluate all sites Venogram is still the gold standard
Cardiac	Right atrial (RA)	CVL malfunction, sepsis, congestive heart failure	ECHO
CVL related	Asymptomatic CVL tip thrombi **ONLY if** the catheter tip is in RA	-	ECHO Linogram Venogram
	**Only** symptomatic CVL TE will be considered significant	Swelling, pain, tenderness, erythema, dilated vessels, CVL malfunction requiring revision or renewal, headache, swelling of face	Linogram +/− venogram &/or ^a^Doppler USG depending upon the site of thrombosis

In the presence of TE at one site recommend evaluating other sites (especially if anatomically related e.g. jugular vessels in presence of SVT) for associated asymptomatic TE, if possible

*TE* thromboembolism, *CNS* central nervous system, *MRI* Magnetic resonance imaging, *MRV* Magnetic resonance venogram, *MRA* Magnetic resonance arteriogram, *PE* pulmonary embolism, *V/Q* scan ventilation/perfusion scan *CT* computerized tomogram, *DVT* deep venous thrombosis, *USG* ultrasonogram, *CVL* central venous line

^a^Detection of echogenic material within the lumen of a vein on a gray scale and presence of partial or complete absence of flow by pulse wave or color Doppler ultrasonography

#### Independent variables

1. Presence of prothrombotic defects: Following specific prothrombotic defects were studied: deficiency of AT, PC, PS; elevated levels of coagulation factors II, VIII, IX and XI; FVL; PT and MTHFR gene mutation; elevated levels of Lp (a), Hcy and APLA
2. Presenting laboratory features (Complete blood count, total white cell count, peripheral blast count, hemoglobin, platelet count)
3. Age of the patient at the time of diagnosis of ALL (age ≥ 10 years, age < 10 years)
4. Risk categorization of ALL (HR/VHR, SR)
5. Therapy Variables: Type of ASP (*E. coli*, PEG ASP)
6. Phase of therapy: Consolidation II versus other phases

### Data collection

#### Clinical data

Specific case report forms (CRF) were created for data collection for all patients. In summary, data included baseline patient characteristics, personal and family history of thrombosis or prothrombotic disorder; any known risk factors (smoking, dyslipidemia, thrombophilia), details of CVL, diagnosis of ALL and ALL-therapy.

#### Laboratory data

Blood samples were tested for complete blood count (CBC), coagulation parameters (INR, PTT, fibrinogen and D-dimer) and prothrombotic defects. For the purpose of this study the prothrombotic defects were divided as follows: **“Variable” defects:** defects in those hemostatic proteins the levels of which are influenced by the exogenous factors like inflammation or anti-leukemic therapy (e.g. ASP and steroids). Deficiency of AT, PC, PS; elevated levels of coagulation factors II, VIII, IX and XI; elevated levels of Lp (a), Hcy and APLA were considered as “variable” defects. **“Non-variable” defects:** those prothrombotic defects, the estimation of which is unlikely to be affected by exogenous factors. These defects include FVL, PT and MTHFR gene mutation. Details of wild type, heterozygous and homozygous state will be collected.

#### Diagnosis, evaluation and management of patients with symptomatic TE

Symptomatic TE was a prospectively defined adverse outcome on DFCI ALL 05–01 study. To ensure uniformity of diagnosis and evaluation, uniform guidelines for the definition of symptomatic TE and clinical and radiological assessment at the time of diagnosis of TE were used as outlined in Table 2. At the time of diagnosis of TE, laboratory evaluation included CBC, coagulation profile (INR, APTT, D-dimer fibrinogen) and measurement of “variable” prothrombotic defects. Patients who develop symptomatic TE were managed according to the recommended uniform guidelines developed for management of TE for DFCI ALL 05–01 study. Details of clinical, laboratory and imaging studies, management and, outcome of TE were recorded on the CRF.

### Assays for hemostatic factors

#### Timing of blood test

Blood samples for prothrombotic defects were collected for all patients prior to starting ALL therapy. For Hcy and Lp(a) estimation overnight fasting blood sample were used. Testing for prothrombotic defects were done on the day of the scheduled lumbar puncture avoiding additional fasting.

#### Sample collection

Detail blood sample collection and processing protocol was developed and provided to the study sites. In short, blood was collected into one 3.2% Sodium citrate tube (~ 1.8 mL) and one EDTA tube (~1.8 mL). Within 30 min of collection, blood was centrifuged to separate platelet poor plasma which was aliquoted and frozen at –70 °C until the time of assay. Citrated sample was used for measurement of factors II, VIII, IX and, XI; AT, PC, and PS; D-dimer, Lp(a) and APLA. EDTA sample was used for Hcy assay. Buffy coats will be used for DNA analysis.

#### Laboratory assays

To avoid inter-laboratory variation, all samples for prothrombotic defects were assayed at centralized location namely the Hemostasis Reference Laboratory (HRL) at Henderson Research Center, Hamilton using standardized procedures http://www.hemostasislab.com. Blood samples collected from patients at other centers were couriered on dry ice to HRL. In addition, CBC, differential, peripheral blast count, and coagulation parameters (PT, APTT, fibrinogen) were performed at individual institution. Samples for prothrombotic defects were assayed in batches.

### Clinical and laboratory follow-up, study duration and study withdrawal criteria

#### Clinical follow up

After initial hospitalization for evaluation and induction therapy for ALL, all patients were seen at least weekly (or more frequently if needed) at the outpatient clinics as a part of routine clinical care. This study required no visits above and beyond those required for this clinical care. Details of hospitalization and other complications including TE during the study-period and modification of ALL-therapy, if any, were recorded. Patients were monitored for failure of remission induction, recurrence of ALL, stem cell transplantation and continued enrollment on DFCI 05–01 study. The clinical follow-up and data collection for the proposed study was linked to DFCI ALL 05–01 study monitoring and follow up.

#### Study duration

Patients were followed for development of symptomatic TE till the completion of ALL-therapy on DFCI 05–01 study which is two years and one month.

#### Study withdrawal criteria

Patients were withdrawn from thrombophilia study if they failed to achieve remission, develop recurrence of ALL while on ALL-therapy, if they needed stem cell transplantation or were withdrawn from DFCI 05–01 study for any reason.

### Time frame of the study

The anticipated total study period was four years. During first two years of the study, patient enrollment including initial data collection and blood sampling were planned to be completed. Patient follow-up continued at respective center till the completion of ALL-therapy (~ 2 years) as scheduled on DFCI 05–01 study.

### Outcomes

#### Primary outcome

Diagnosis of symptomatic TE in any location while on active ALL-therapy.

#### Secondary outcomes

Includes detection of at least one prothrombotic defect, completion of ALL-therapy or withdrawal from DFCI 05–01 study and recurrence of disease or death due to any cause while on DFCI 05–01 ALL-therapy.

#### Justification for inclusion of symptomatic TE

Clinical significance of asymptomatic TE detected by screening methods is so far unknown. Further, by inclusion of only symptomatic and objectively confirmed TE, we will avoid ambiguity over diagnosis, and thus, reporting of TE. Also the inclusion of only symptomatic TE will avoid invasive (e.g. venography) and non-invasive (e.g. ultra-sonography) tests to screen for asymptomatic TE. This, we hope, will improve patient and physician compliance and participation in the study.

#### Criteria for diagnosing symptomatic TE

Objective testing must be done to confirm suspected thrombotic events in symptomatic patient (Details in Table 2). Acceptable objective tests for diagnosis of CNS TE: cerebral angiographies, contrast enhanced magnetic resonance imaging (MRI), MR arteriography (MRA) or MR venography (MRV); deep venous thrombosis (DVT) include: venography, Doppler ultrasonography (USG), contrast enhanced computerized tomography (CT), MRV; for PE: pulmonary angiography, ventilation/perfusion (V/Q) scan or spiral CT scan; and for right atrial TE: echocardiography (ECHO). DVT was diagnosed by detection of echogenic material within the lumen of a vein on a gray scale and presence of partial or complete absence of flow by pulse wave or color Doppler ultrasonography or if venography or contrast enhanced CT shows a persistent intraluminal filling defect [9, 56, 57]. CNS-TE was diagnosed if imaging modalities showed intraluminal arterial or venous filling defect/s with or without associated cortical changes. PE was confirmed with intraluminal filling defect or abrupt cut-off or non-filling of an arterial segment on either pulmonary angiograpghy or spiral CT scan or a high probability V/Q scan.

#### Criteria for diagnosing prothrombotic defect

Age-adjusted standardized laboratory data were used for classification of protein deficiencies (PC, PS, AT) or elevation (F II, VIII, IX, XI) and risk cutoffs [Lp (a), Hcy, APLA]. For this purposes the age-adjusted laboratory normal values based on Canadian population data were used [58]. The actual value was categorized as deficiency, normal range or elevation of the factor studied. Identification of the gene mutation will confirm FVL, PT and MTHFR polymorphism.

### Statistical considerations

#### Study variables

Continous variables will be age, levels of factors II, VIII, IX and XI; PC, PS, AT; Lp (a), and Hcy. Categorical variables will be gender (male/female); ALL risk categories (VHR/HR/SR); ASP type (*E. coli*/PEG); details of CVL (presence/absence),“non-variable” prothrombotic defects (FVL, PT and MTHFR mutation) (presence/absence).

#### Methods for analysis

Patient demographics, prognostic characteristics and clinical outcome will be summarized using descriptive measures expressed as mean (standard deviation) or median (minimum, maximum) for continuous variables and number (percent) for categorical variables. The analysis will adopt the intention-to-treat principle. Since TE is a safety outcome for ALL-therapy we will also perform “per-protocol” analysis. Association between categorical outcomes and groups will be assessed using Fisher’s Exact test or chi-squared test. Thrombosis-free survival will be estimated using Kaplan-Meier method. The limit for statistical significance will be set at α = 0.05. In all comparison, 95% confidence intervals (CI) of the measure of association between groups will be reported.

Logistic regression will be used to analyze data for both primary and secondary outcomes. Analysis results of regression modeling will expressed as coefficient, corresponding standard error, 95% CI and associated *p*-values. Variance inflation factors will be used to assess multi-colinearity among predictors. Model assumptions will be assessed through residual analysis. Goodness-of-fit will be evaluated using qq plots for normality and coefficient of determination and R^2^ for regression models. Some of the time dependent confounding variables like infection or CVL-related problems (which may influence the risk of TE) are unpredictable and hence cannot be adjusted prospectively. To compensate for that we will prospectively collect the information and enter in the regression model when risk factors for thrombosis are being analyzed. All analyses will be performed using SPSS 14 software (2005 SPSS Inc). Table 3 provides a summary of specific methods of analysis for primary and secondary outcomes.

**Table 3:** Proposed Methods of Analysis

Analysis	Hypothesis	Independent variable	Outcome variable		Method of analysis
			Name	Variable type	
Primary	Presence of one or more prothrombotic defect/s increase the risk of TE	Presence of one or more prothrombotic defect	Symptomatic TE	Binary	Fisher’s Exact Test Analysis performed for overall prevalence of at least one prothrombotic defect and for individual defect.
Secondary					
Aim 1.	1.a Older age increases the risk of TE, especially in presence of one or more prothrombotic defect	Age of the patient (age < 10 years, age ≥ 10 years)	Symptomatic TE	Binary	Logistic regression
	1.b HR/VHR ALL increases the risk of TE, especially in presence of one or more prothrombotic defect	Risk categorization of ALL (HR/VHR ALL, SR ALL)			
	1.c PEG ASP therapy increases the risk of TE, especially in presence of one or more prothrombotic defect	Type of ASP (*E. coli* ASP, PEG ASP)			
Aim 2	A mathematical model can be used to determine the risk of symptomatic TE	Clinical Variables: age of the patient, risk categorization of ALL presence or absence of prothrombotic defect, Therapy variable: type of ASP used	Symptomatic TE	Binary	Regression analysis will be used to predict the risk of TE
Aim 3	The overall (presence of at least one tested) prevalence of one or more prothrombotic defect is at least 20%		Prevalence of one or more prothrombotic defect/s	Categorical	Prevalence of individual and overall prothrombotic defect will be expressed as percentage of affected patients with 95% CI

Logistic regression will be used to analyze the data for both primary and secondary outcomes. Analysis results of regression modeling will be expressed as coefficient, corresponding standard error, 95% CI and associated *p*-values. Variance inflation factors will be used to assess multi-collinearity among predictors. Missing values will be handled using multiple imputation or last observation carried forward. Model assumptions will be assessed through residual analysis. Goodness-of-fit will be evaluated using qq plots for normality and coefficient of determination and R2 for regression models

#### Sample size

The sample size calculation is based around the primary aim of the study. The null hypothesis for the primary aim of this study that there is no difference in the proportion of patients developing TE with or without prothrombotic defect. The criterion for significance is set at two sided α = 0.05. *Minimal clinically important difference (MCID):* The purpose of this study is to identify a population at high risk for TE, so that we can develop strategies for prevention of TE. The MCID we wish to detect will be guided by the numbers needed to treat (NNT) to prevent one event of TE. If we have to use presence of prothrombotic defect as the only criteria for effective thromboprophylaxis, we aim to detect a MCID of at least 25%. This will mean that, we will need to treat ~4 patients to prevent one event of TE. Table 4 outlines the required sample size calculation and NNT at various levels of MCID. Based on the preliminary data from Canadian institutions on previous DFCI protocol 20–01, the incidence of TE is estimated to be 11%. Based on previous studies, the overall prevalence of prothrombotic defect (presence of at least one prothrombotic defect) in the study cohort is estimated to be 20% [38, 39, 48]. With a sample of 150 patients (30 patients with and 120 without prothrombotic defect) there is more than 80% chance of detecting MCID of 25% at a two-sided significance level of 0.05.

**Table 4:** Sample size calculation requirements to compare cumulative TE in patients with and without prothrombotic defects

δ	Sample size for each group	Estimated sample size for proposed study (α =0.05 and β = 0.2, with 20% prevalence of prothombotic defects based on previous studies [12])			Number of patients needed to treat to prevent one event of TE (NNT)
		With PD	Without PD	Total	
0.40	16	16	80	96	approximately 3 patients
0.30^a^	24	24	120	144	approximately 4 patients
0.20	41	41	205	246	5 patients
0.10	102	102	510	612	10 patients

^δ^Difference in the cumulative incidence of TE in patients with and without prothrombotic defects

^a^Minimal clinically important difference

#### Feasibility

At three participating institutions, every year about 90 children are diagnosed with ALL. Our experience on previous protocols indicate almost 100% patient participation in DFCI ALL studies. However, accounting for ineligibility or non-enrollment either on DFCI 05–01 study (~5%) or the proposed thrombophilia study (~5%) and withdrawal from DFCI 05–01 study (~5%) we anticipate a sample size of at least 150 patients over the period of 2 years. Based on the previous experience of age distribution and risk-stratification, we anticipate that about 26 patients will be 10 years or older and ~80 patients will be classified as HR/VHR ALL in the study cohort of 150 patients. With 17% incidence of TE in HR population and about 2% in SR population based on our previous analysis, we anticipate about 14 patients with TE in HR/VHR group and 2 patients with TE in the SR group. All three centers have the necessary laboratory and technical support for investigational blood collection. Hence, the proposed study is feasible to address the primary aim.

#### Limitations

The sample size is calculated on the presumption of 20% overall prevalence of prothrombotic defect in children with ALL based on previous studies [38, 39, 48]. If this presumption fails then the sample size may not be adequate to answer the primary aim. *Back up plan for patient enrollment and completion of the study:*The Consortium-wide DFCI-05-01 RCT will enroll about 550 patients over a period of 5 years (from June 2005–May 2010) from nine institutions of the Consortium. Although we are confident of enrolling the required sample size from participating institutions, in the event of unforeseen problems with patient enrollment or lower prevalence of prothrombotic defects in North American children, we could extend the duration of study period and/or expand the study to other institutions within the DFCI Consortium. This will enable us to achieve adequate patient enrollment to address the primary aim of the proposed thrombophilia study.

### Validity of the study

#### Internal validity of the proposed study

The following steps will be taken to minimize sources of errors and likely biases: *1) Sampling:* To avoid selection bias all newly diagnosed ALL patients who are enrolled on DFCI 05–01 study at three participating institutions will be eligible for proposed thrombophilia study. *2) Measurement of outcomes and variables:* a) Primary outcome:By inclusion of only symptomatic and objectively confirmed TE, we will avoid ambiguity over diagnosis, and thus, reporting of TE. Uniform guidelines for diagnosis and evaluation will likely minimize chances of misdiagnosis of TE. All suspected events of TE will be confirmed by independent adjudication committee. b) Measurement of Variables: Laboratory assays for prothrombotic defects for patients will be performed at a central laboratory, thus, avoiding inter-laboratory variation. *3) Data collection and analysis:* With the use of uniform data collection forms for all study participants and patients with TE all the relevant information is likely to be captured. Teleform method will be used for data entry which will minimize errors. Further, all data will be analyzed centrally.

#### Generalizability

The results of this study will be applicable to future DFCI ALL study protocols since the basic design of DFCI studies (including drug combination, delivery sequence and dosage) is unchanged over the years [52]. Since most frontline ALL-therapy protocols use ASP and steroids, the results of the proposed study will also be applicable to children and adults with ALL treated on other protocols. Further, some of the results will be easily generalized, for example prevalence of prothrombotic defects in North American children with ALL.

### Ethical considerations

The study was presented to the institutional review board (IRB) of each participating institution and the IRB approval was obtained from the Hamilton Health Sciences/McMaster University Faculty of Health Science Research Ethics Board (project # 06–192) and the Comité d’Éthique à la recherche du CHU Sainte-Justine (project # 2609).

#### Informed consent

Patients were enrolled on the study only after the informed consent has been obtained from the patient or the parent/guardian according to the IRB guidelines. In addition, **assent** was obtained for patients between the ages of 12–16 years (or younger if perceived competent to do so by physicians) as per institutional guidelines.

#### Patient safety and inconvenience

This study does not pose any additional risks to the patients. The amount of blood drawn was very minimal (~ 4 mL) and drawn in conjunction with other tests required for evaluation and standard care of the patients, thus avoiding additional inconvenience to the patient. Similarly fasting blood tests were performed on the days of fasting for therapeutic procedures avoiding extra fasting.

#### Confidentiality

*A*ll the data will be coded and stored securely to protect individual confidentiality.

#### Patient care and benefit

Patients with TE were evaluated, treated, and counseled according to the recommended standard of care. Patients diagnosed with prothrombotic defects, with or without TE, will receive appropriate counseling.

## Discussion

So far the most acceptable way to prevent TE in a population with cancer is daily subcutaneous low molecular weight heparin (LMWH) injections [59]. The primary thromboprophylaxis studies in adult cancer patients have shown relative risk reduction in the range of 40–50% [actual risk reductions 13–17% (22% to 9% and 30% to 13%)] in both surgical and medical oncology patients [59]. Based on these studies, primary prophylaxis with LMWH is now a standard of care in adult cancer patients who are at high risk for TE [60]. However, thromboprophylaxis is not yet a standard of practice in majority of high-risk pediatric population. There are no randomized controlled trials (RCT) of primary or secondary thromboprophylaxis in children with cancer. The inherent risk of bleeding in children with ALL discourages the use of thromboprophylaxis. Only one study has used primary LMWH prophylaxis in children receiving ASP on Israeli BFM ALL 90/95 protocol (*n* = 41; 43% with thrombophilia) [49]. Compared to the historic control (48%) they report no TE in children with thrombophila [38, 49]. None of the patients on LMWH prophylaxis had a bleeding episode. Although this is a small observational study, it shows that LMWH can be safe and effective in preventing TE in children with ALL.

Over 50% of symptomatic TE in children with ALL occur in CNS; majority of these events are venous in the form of cerebral sinovenous thrombosis (CSVT). Observational studies in children with non-caner associated strokes have shown complete resolution of TE with anticoagulant therapy with either unfractionated heparin (UFH) or LMWH [56]. *Chest* guidelines recommend antithrombotic therapy for treatment of pediatric stroke and CSVT [56, 61]. LMWH has been successfully used in the treatment and secondary prevention of CSVT in children with ALL [62]. Thus, LMWH prophylaxis is likely to be useful to prevent CNS-TE in children with ALL.

Further studies are certainly warranted to evaluate the role of thromboprophylaxis and the ideal agent for such prophylaxis in children with ALL. However, the first step is to identify children with ALL who are at high risk for TE which is the objective of present study.

To our knowledge, this will be the first prospective and comprehensive evaluation of risk factors for development of TE conducted in a large cohort of North American children treated on a uniform ALL-therapy protocol. This study will be important to define the epidemiology and to identify the factors predisposing to thrombosis in children treated on DFCI ALL therapy protocols. In addition, the proposed study will define the prevalence of wide range of prothrombotic defects in North American children with ALL. The results of this study will be used to design future randomized controlled trial of prophylactic anticoagulant therapy to reduce the incidence of TE in children with ALL in the context of next DFCI ALL study protocol. This will ultimately help to reduce the incidence of TE and its impact on overall outcome as well as quality of life in children undergoing treatment for ALL.

### Study progress and modification to study protocol

The study is currently in follow-up phase. This study highlights the problems of conducting a prospective, observational studies in pediatric oncology patient population.

1. Extension of study duration: Due to logistics issue the study could not be activated at third clinical site. This created challenges in patient enrolment. Hence the total duration of patient enrolment from two sites was extended.
2. Addition of blood group information to variables to be collected: During the course of the study, additional information from a local study from Ste. Justine site became available which showed impact of blood groups on the risk of TE (*Mizrahi T. Personal communication*). In this retrospective study of children treated on DFCI ALL Consortium therapy protocols (*n* = 523), non-O blood group was identified as an independent risk factor. Hence after study amendment and REB approval, data regarding ABO blood group was collected on all participants. Since almost all children get blood transfusion during the course of chemotherapy, the data regarding ABO blood group is easily available. This also resulted in modification of analyses plan. The ABO blood group will be added as a categorical variable (non-O vs. O blood group) and tested in univariate and multivariate analyses.
3. Preliminary data analyses showed 15% cumulative incidence of TE and high prevalence of prothrombotic defects in the study population. This allowed us to reduce the required sample size to 131. This was especially important since patient enrolment was very slow.

## Abbreviations

ALL: Acute lymphoblastic leukemia
APLA: Antiphospoholipid antibodies
ASP: Asparaginase
AT: Antithrombin
BFM: Berlin-Frankfurt-Münster
CBC: Complete blood count
CI: Confidence intervals
CNS: Central nervous system
CSVT: Cerebral sinovenous thrombosis
CT: Computerized tomography
CVL: Central venous line
DFCI: Dana-Farber cancer institute
DVT: Deep venous thrombosis
ECHO: Echocardiography
FVIII:C: Coagulation factor VIII:C
FVL: Factor Vleiden
Hcy: Homocysteine
HR: High risk
HRL: Hemostasis reference laboratory
IRB: Institutional review board
LMWH: Low molecular weight heparin
Lp(a): Lipoprotein (a)
MCID: Minimal clinically important difference
MRA: Magenetic resonance arteriography
MRI: Magnetic resonance imaging
MRV: Magnetic resonance venography
MTHFR: Methylene tetrahydrofolate reductase
NNT: Number needed to treat
PAI1: Plasminogen activator inhibitor 1
PARKAA: Prophylactic antithrombin replacement in kids with acute lymphoblastic leukemia treated with asparaginase
PC: Protein C
PE: Pulmonary embolism
PEG: Pegaspargase
PS: Protein S
PT: Prothrombin
RCT: Randomized controlled trial
SR: Standard risk
TE: Thromboembolism
USG: Doppler ultrasonography
V/Q: Ventilation/perfusion scan
VHR: Very high risk
